# Mechanism of Thiosulfate Oxidation in the SoxA Family of Cysteine-ligated Cytochromes

**DOI:** 10.1074/jbc.M114.618025

**Published:** 2015-02-11

**Authors:** Daniel B. Grabarczyk, Paul E. Chappell, Bianca Eisel, Steven Johnson, Susan M. Lea, Ben C. Berks

**Affiliations:** From the ‡ Department of Biochemistry, University of Oxford, South Parks Road, Oxford OX1 3QU, United Kingdom and; the § Sir William Dunn School of Pathology, University of Oxford, South Parks Road, Oxford OX1 3RE, United Kingdom

**Keywords:** Bacterial Metabolism, Cytochrome, Electron Transport, Heme, Metalloenzyme, Sox System, Sulfur Oxidizing Bacteria, Thiosulfate Dehydrogenase

## Abstract

Thiosulfate dehydrogenase (TsdA) catalyzes the oxidation of two thiosulfate molecules to form tetrathionate and is predicted to use an unusual cysteine-ligated heme as the catalytic cofactor. We have determined the structure of *Allochromatium vinosum* TsdA to a resolution of 1.3 Å. This structure confirms the active site heme ligation, identifies a thiosulfate binding site within the active site cavity, and reveals an electron transfer route from the catalytic heme, through a second heme group to the external electron acceptor. We provide multiple lines of evidence that the catalytic reaction proceeds through the intermediate formation of a *S*-thiosulfonate derivative of the heme cysteine ligand: the cysteine is reactive and is accessible to electrophilic attack; cysteine *S*-thiosulfonate is formed by the addition of thiosulfate or following the reverse reaction with tetrathionate; the *S*-thiosulfonate modification is removed through catalysis; and alkylating the cysteine blocks activity. Active site amino acid residues required for catalysis were identified by mutagenesis and are inferred to also play a role in stabilizing the *S*-thiosulfonate intermediate. The enzyme SoxAX, which catalyzes the first step in the bacterial Sox thiosulfate oxidation pathway, is homologous to TsdA and can be inferred to use a related catalytic mechanism.

## Teaser

**Background:** The hemoprotein TsdA catalyzes the oxidation of two thiosulfate molecules to form tetrathionate.

**Results:** The mechanism of TsdA has been probed using biochemical and structural methods.

**Conclusion:** The TsdA reaction proceeds via a cysteine *S*-thiosulfonate intermediate formed on a cysteine ligand to the active site heme.

**Significance:** TsdA provides a catalytic model for other members of the SoxA enzyme family.

## Introduction

Thiosulfate is an important intermediate in the biogeochemical sulfur cycle. It is formed by oxidation of sulfide in soils and sediments (1) and is also the product of sulfide detoxification in the animal gut (2).

Many sulfur bacteria use thiosulfate either as a source of reductant for carbon dioxide fixation or as the electron donor to energy-generating respiratory electron transport chains. The widely distributed Sox pathway allows these bacteria to oxidize thiosulfate completely to sulfate (3–5). Alternatively, some sulfur bacteria can oxidize thiosulfate to tetrathionate using thiosulfate dehydrogenase (TsdA)^6^ (6, 7).

The first step in the Sox thiosulfate oxidation pathway involves formation of a disulfide bond between thiosulfate and a cysteine residue on the sulfur carrier protein SoxYZ (Reaction 1).

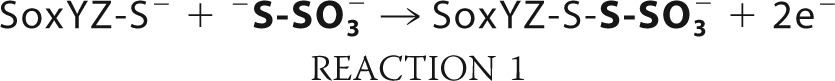
 This reaction is catalyzed by the heterodimeric *c*-type cytochrome SoxAX (8). The active site in this enzyme is located in a heme-containing domain of the SoxA subunit. Structural analysis shows that the substrate-accessible side of the active site heme has an unusual protein ligand: a cysteine residue that has been modified by the addition of a terminal sulfur atom to form cysteine *S*-sulfane (8–11). Although a few other *c*-type cytochromes have cysteine-coordinated hemes (12–16), SoxA is unique in having a cysteine *S*-sulfane adduct. This has led to the suggestion that the ligand is an active participant in the catalytic chemistry of the enzyme (9). Specifically, it has been proposed that catalysis proceeds in two steps via an *S*-thiosulfonate adduct of the cysteine ligand (Reactions 2 and 3).

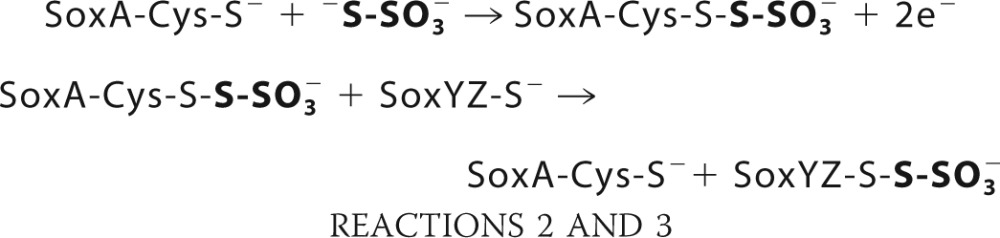
 In the first step, thiosulfate is oxidatively conjugated to the active site cysteine residue. The resultant *S*-thiosulfonate adduct is resolved in the second step by a thiol-disulfide exchange reaction with the reactive cysteine of the SoxYZ carrier protein. The cysteine *S*-sulfane adduct seen in the purified SoxAX enzyme is assumed to arise from an off-pathway reaction of the cysteine *S*-thiosulfonate intermediate (9). Unfortunately, SoxAX has proved highly recalcitrant to mechanistic analysis over many years, so there is currently no evidence to support the proposed cysteine-mediated reaction mechanism. Indeed, it has recently been claimed that SoxAX is still enzymatically active after substitution of the heme cysteine ligand, and an alternative mechanism involving a copper ion has been put forward (11).

Thiosulfate oxidation by the thiosulfate dehydrogenase TsdA involves the oxidative conjugation of two thiosulfate molecules to form tetrathionate (Reaction 4) in a reaction analogous to the one catalyzed by SoxAX (Reaction 1).

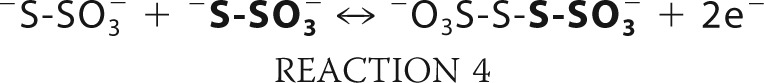
 Intriguingly, the N-terminal half of *Allochromatium vinosum* TsdA was recently found to be homologous to the SoxA catalytic domain (7), suggesting that TsdA and SoxAX have related mechanisms. This is an exciting observation because it opens up the possibility of using TsdA as an experimentally accessible model for understanding SoxA catalysis. Sequence analysis identifies Cys^123^ as the ligand to the *A. vinosum* TsdA catalytic heme, and mutagenesis shows this residue to be essential for enzyme activity (7). A two-step model for thiosulfate oxidation by TsdA can be proposed by analogy to the putative SoxAX mechanism, with the second molecule of thiosulfate taking the place of SoxYZ (Reactions 5 and 6) (7).

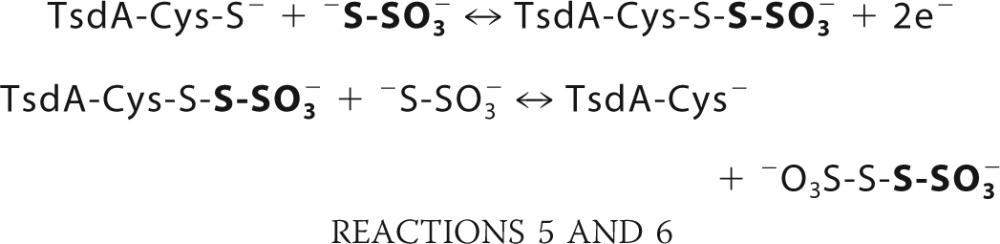
 In the presence of a suitable electron donor TsdA enzymes are able to reverse the normal catalytic reaction and reduce tetrathionate to two molecules of thiosulfate, although for the *A. vinosum* enzyme this reaction is much slower than the forward reaction (17).

Using the TsdA enzyme of *A. vinosum* as our model, we present biochemical evidence that supports the involvement of an *S*-thiosulfonate intermediate in the reactions catalyzed by SoxA family enzymes. We also use structural analysis and mutagenesis to identify active site residues involved in substrate binding and catalysis.

## EXPERIMENTAL PROCEDURES

### 

#### 

##### Genetic Constructs

A construct to express *A. vinosum* TsdA protein with its native signal peptide and a C-terminal strep tag (18) was constructed as follows. The *tsdA* gene was amplified from *A. vinosum* genomic DNA using primers 5′-CAAGACTCTAGAATGCGCGGTGATGTAAGG-3′ and 5′-ACTTACTCTAGATCAGTCACCCGTCGGC-3′, cut with XbaI, and cloned into the XbaI site of plasmid pTZ19R (19). The cloned *tsdA* gene was reamplified using primers 5′-CAGGACGAATTCATTAAAGAGGAGAAATTAACTATGCGCGGTGATGTAAGG-3′ and 5′-ACTTACAAGCTTTCATTTTTCGAACTGCGGGTGGCTCCAGCTAGCGTCACCCGTCGGCTC-3′. These introduce an optimized *E. coli* ribosome binding site upstream of the gene and fuse a strep(II) tag-coding region to the end of *tsdA*, respectively. The resulting amplicon was digested with EcoRI and HindIII and used to replace the EcoRI-HindIII fragment of plasmid pQE80L (Qiagen) to give plasmid pQE80L-AvTsdA-strep.

##### Purification of TsdA

*Escherichia coli* strain Lemo56(DE3) (20) was co-transformed with plasmid pQE80L-AvTsdA-strep and plasmid pEC86 (21), which constitutively expresses the *E. coli* cytochrome *c* maturation system. Cells were grown aerobically at 30 °C in LB medium (22) supplemented with 100 μg/ml ampicillin and 25 μg/ml chloramphenicol. Protein expression was induced at an *A*_600 nm_ = 0.6 with 1 mm isopropyl-1-thio-β-d-galactopyranoside, and the cells were cultured for a further 16 h at 20 °C.

Cells were harvested by centrifugation and resuspended in resuspension buffer (100 mm Tris-HCl, pH 8.0, 200 mm NaCl, 1 mm EDTA), together with EDTA-free complete protease inhibitors (Roche Applied Science) and a few crystals of lysozyme and DNase I (both from Sigma-Aldrich). The cells were broken by three passages through a French press at 8000 p.s.i. The lysate was clarified by centrifugation at 200,000 × *g* for 1 h at 4 °C and then applied to a 5-ml self-packaged Strep-Tactin column (IBA, GmbH, Göttingen, Germany). Recombinant TsdA was eluted from the column with resuspension buffer containing 2.5 mm desthiobiotin. The eluate was diluted 5-fold in water to reduce the ionic strength of the buffer and subjected to ion exchange chromatography on a MonoQ HR 5/5 column (GE Healthcare) using a 0–500 mm linear NaCl gradient in 30 mm Tris-HCl, pH 8.0, buffer. TsdA was further purified on a Superdex75 (16/60) preparative grade size exclusion column (GE Healthcare) that had been equilibrated in 30 mm Tris-HCl, pH 8.0, 160 mm NaCl. TsdA concentrations were determined from the heme concentration measured by the pyridine hemochrome method (23).

##### Mass Spectrometry

Samples for analysis (typically 20 μl) were diluted 1:50 in 0.1% (v/v) trifluoroacetic acid (TFA) to stop further enzymatic reactions and then desalted into 50% (v/v) acetonitrile/water, 0.1% formic acid using in-line reverse phase chromatography before loading on a Waters LCT Premier electrospray ionization mass spectrometer operated in positive ion mode. In preliminary experiments we noticed that TsdA samples with reduced hemes were not detected under our electrospray ionization mass spectrometry (ESI-MS) conditions. Consequently, we treated all samples containing reduced TsdA with a 10-fold molar excess of potassium ferricyanide immediately before the desalting step to oxidize the hemes. All spectra were acquired and analyzed using MassLynx software (Waters).

##### Activity Assay

Thiosulfate dehydrogenase activity was assayed using ferricyanide as an electron acceptor. Unless otherwise indicated, assays were carried out at 24 °C in 100 mm ammonium acetate, pH 4.25, 8 mm sodium thiosulfate and were initiated by the addition of 1 mm potassium ferricyanide. Activity was quantified from the decrease in absorbance at 420 nm using Δϵ_420 nm_ (ferricyanide-ferrocyanide) = 1.09 mm^−1^ cm^−1^.

##### Protein Crystallization, X-ray Data Collection, Structure Solution, and Refinement

TsdA was crystallized using the sitting drop, vapor diffusion method. 200-nl drops containing 70% protein solution (380 μm TsdA and 2 mm sodium tetrathionate in 10 mm Tris-HCl, pH 8.0) and 30% mother liquor (100 mm ammonium acetate, pH 4.6, 7.5% PEG 10,000) were equilibrated against 120 μl of mother liquor at 20 °C. Crystals, which grew in 2 weeks, were cryoprotected in 30% ethylene glycol, 70% mother liquor and flash-cooled in liquid nitrogen. Diffraction data were collected to 1.29 Å at 100 K on the i03 beamline at the Diamond Light Source (Oxfordshire, UK). Automatic data processing was carried out with the Xia2 package (24). Molecular replacement was carried out using MolRep (25) with search models derived from the C-terminal domain of *R. sulfidophilum* SoxA (PDB entry 1H32) and the C-terminal region of *Pseudoalteromonas haloplanktis* nitrite reductase (PDB entry 2ZOO). Autobuilding was carried out with ARP/wARP (26), and subsequent refinement and rebuilding cycles were carried out using Coot (27) and Refmac (28). Residual log-likelihood gradient maps were calculated using external phases from the final model in data collected at λ = 1.4 Å within SHARP (29). The model phases were combined within SHARP with phases derived by explicit modeling of the iron atoms within the heme groups to allow clear visualization of the scattering from the sulfur atoms. Simulated annealing omit maps were calculated using Phenix (30) to confirm modeling of the active site. Details of data collection and data quality are given in Table 1. Structural figures were produced using the PyMOL Molecular Graphics System, version 1.3 (Schrödinger, LLC).

## RESULTS

**FIGURE 1.**
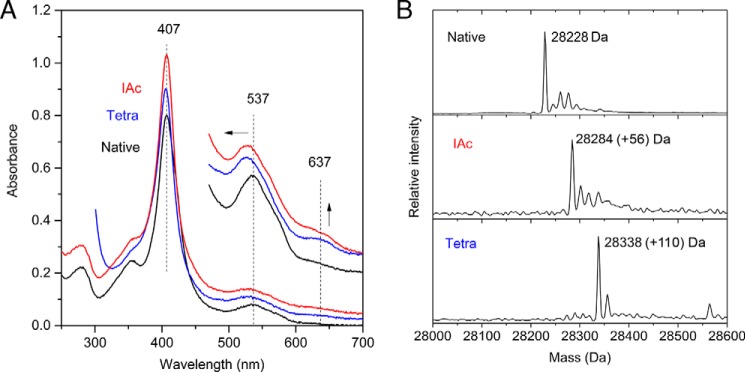
**Modification of Cys^123^ by electrophiles at pH 8.** *A*, UV-visible spectra of 4 μm (*main spectra*) or 20 μm (*high wavelength inset*) TsdA in 30 mm Tris-HCl, pH 8.0, 160 mm NaCl. Samples were either untreated (*Native*), incubated for 1 h with 50 mm sodium tetrathionate (*Tetra*), or incubated for 1 h with 20 mm sodium iodoacetate followed by desalting by size exclusion chromatography (*IAc*). Spectra are presented offset in the *y* axis direction for clarity. *Arrows* indicate the movement or intensity change of the highlighted peaks. *B*, ESI-MS analysis of the samples from *A*. The expected mass increases for thiosulfonation and for carboxymethylation are 112 and 57 Da, respectively.

We have used recombinant *A. vinosum* TsdA to investigate the mechanism of thiosulfate dehydrogenase. In this protein, Cys^123^ is predicted to provide the substrate-side ligand to the active site heme iron (7). The only other cysteine residues in TsdA are found in the two heme attachment motifs, where they provide covalent linkages to the heme vinyl groups. ESI-MS of purified TsdA showed a single peak at 28,228 Da (Fig. 1*B*), which corresponds almost exactly to the predicted mass of 28,226 Da calculated for the recombinant protein with two covalently bound *c*-type hemes. Thus, Cys^123^ is unmodified in our TsdA preparation, in marked contrast to preparations of the homologous SoxAX proteins, where both native and recombinant proteins exhibit a *S*-sulfane modification of the active site cysteine (9–11).

### 

#### 

##### Reactivity of the Active Site Cysteine Residue

We have used our TsdA preparation to test the proposal that TsdA catalysis involves formation of an *S*-thiosulfonate adduct on the active site cysteine residue (Reactions 5 and 6). This mechanistic model makes the prediction that the active site cysteine residue is reactive although it is a heme ligand. More specifically, it predicts that the cysteine is able to undertake a nucleophilic attack on tetrathionate when the enzyme is operated in the reverse direction (Reaction 6). For this to be possible, the cysteine must be able to dissociate from the heme iron and, thus, should be accessible to electrophilic thiolate-reactive agents. To test whether this is the case, we incubated TsdA with the thiol-selective alkylating agent iodoacetate. This treatment increased the mass of TsdA by 58 Da, consistent with Cys^123^ undergoing *S*-carboxymethylation (Fig. 1*B*). The UV-visible spectrum of *S*-carboxymethylated TsdA showed three main differences compared with native oxidized TsdA, all of which are characteristic of an increase in the content of ferric high spin *c*-type heme (31) (Fig. 1*A*). First, a broadening of the Soret band at 407 nm to lower wavelengths. Second, a shift of the broad oxidized α and β bands centered around 537 nm to lower wavelength. Third, a large increase in the intensity of the band at 637 nm previously assigned to high spin heme (7). The increased proportion of high spin heme in iodoacetate-treated TsdA suggests that the *S*-carboxymethyl modification either prevents the active site cysteine from coordinating the heme iron or acts as a weak field ligand as seen for His/carboxylate-coordinated hemes (32).

**FIGURE 2.**
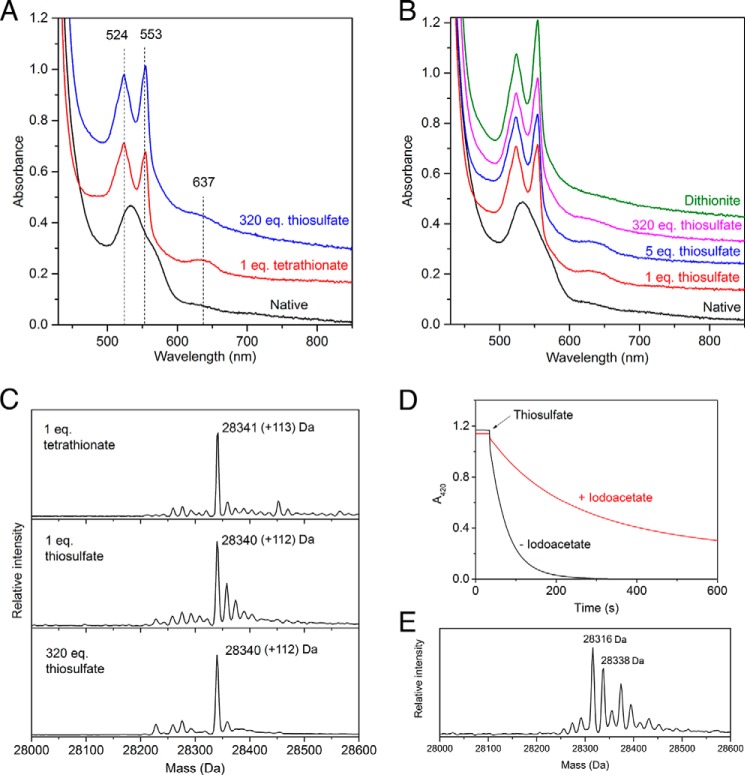
**Reactivity of TsdA with thiosulfate and tetrathionate at pH 4.25.** *A–C*, 25 μm TsdA in 100 mm ammonium acetate, pH 4.25, was either left untreated (*Native*) or incubated for 3 min with the indicated equivalents of either thiosulfate or tetrathionate. *A* and *B*, visible spectra of a single sample to which the indicated additions were made successively in the order *bottom* to *top*. In *B*, dithionite was added until no further spectral changes were seen. For clarity, the spectra are offset relative to the native spectrum. *C*, samples were quenched in 0.1% TFA and subjected to ESI-MS. The expected mass increase for thiosulfonation is 112 Da. *D*, enzyme turnover removes thiosulfonation from Cys^123^. Reactions contained 1 nm *S*-thiosulfonated TsdA and 1 mm potassium ferricyanide in 100 mm ammonium acetate, pH 4.25, either in the presence or absence of 10 mm iodoacetate. The reaction was initiated by the addition of 8 mm thiosulfate, and oxidation of ferricyanide was monitored at 420 nm. *E*, the cysteine *S*-thiosulfonate group formed at the TsdA active site is unstable. Thiosulfonated TsdA was generated by the addition of tetrathionate at pH 8.0 and then desalted into 30 mm Tris-HCl, pH 8.0, 160 mm NaCl. This sample was incubated for 1 h at 37 °C in the presence of 20 mm sodium iodoacetate to capture any thiols formed, quenched in 0.1% TFA, and analyzed by ESI-MS. The expected mass of the starting cysteine *S*-thiosulfonate species is 28,340 Da, and the expected mass of a *S*-carboxymethylated cysteine persulfide adduct is 28,317 Da.

The mechanistic model suggests that the putative cysteine *S*-thiosulfonate intermediate can be generated in the reverse direction by reaction with tetrathionate (Reaction 6) and that in the absence of an electron donor, this intermediate will accumulate and can be characterized. We tested this prediction by incubating TsdA with tetrathionate either at the catalytic pH optimum of 4.25 (7) or at pH 8.0, where the enzyme has little activity (17). Tetrathionate treatment under both conditions gave the 112-Da increase in mass expected for formation of the *S*-thiosulfonate intermediate (Figs. 1*B* and 2*C*) and was able to prevent subsequent alkylation by iodoacetate. Complete *S*-thiosulfonation of the protein took 1 h at pH 8.0 even in the presence of 50 mm tetrathionate, consistent with the low tetrathionate reductase activity of *A. vinosum* TsdA at circumneutral pH (17). By contrast, modification of TsdA by a stoichiometric quantity of tetrathionate was complete within the mixing time of the experiment at pH 4.25, as expected of an enzyme-catalyzed reaction. The visible spectrum of TsdA modified with tetrathionate at pH 8.0 is similar to that of the iodoacetate derivative (Fig. 1*A*), suggesting that carboxymethylation and thiosulfonation of Cys^123^ have similar effects on the active site heme ligation. Tetrathionate treatment at pH 4.25 elicits the same large increase in the intensity of the 637 nm band that is seen at pH 8.0 (Fig. 2*A*). There are also increases in the intensity and sharpness of the heme α and β bands at 553 and 524 nm characteristic of heme reduction. We ascribe the presence of reduced heme to the enzymatic reduction of TsdA by thiosulfate released in the tetrathionate modification reaction (Reaction 6). Presumably, this thiosulfate oxidation reaction is negligible at pH 8.0 for kinetic reasons.

We next tried to generate the proposed cysteine *S*-thiosulfonate intermediate by running the TsdA reaction in the forward direction. Because the steps that produce and resolve the intermediate each involve reaction with one molecule of thiosulfate (Reactions 5 and 6), we attempted to maximize the levels of the intermediate species present by reacting TsdA with only one equivalent of thiosulfate. ESI-MS showed that complete thiosulfonation of the TsdA protein had taken place (Fig. 2*C*). Examination of the visible spectrum revealed that the reaction with thiosulfate had reduced a large proportion of the heme groups present, as anticipated for the first step of the forward reaction (Fig. 2*B*). In addition, there was significant intensity in the 637 nm band originating from the cysteine *S*-thiosulfonate form of the oxidized active site heme. The presence of oxidized heme in the thiosulfonated enzyme is probably due to electron redistribution among the TsdA molecules. Taken together, these data suggest that the majority of the TsdA molecules in the sample had reacted with one molecule of thiosulfate to form a *S*-thiosulfonate derivative. The proposed catalytic model predicts that the further addition of thiosulfate molecules would result in the removal of the *S*-thiosulfonate group as tetrathionate (Reaction 6). However, TsdA remained fully thiosulfonated even after the addition of a considerable molar excess of thiosulfate (Fig. 2*C*), although some further heme reduction took place (Fig. 2*B*). One possible explanation for this unexpected result would be that the second step of the reaction cannot occur until the active site heme is reoxidized. However, this observation also left open the possibility that the cysteine *S*-thiosulfonate adduct is not an authentic pathway intermediate. We therefore examined whether the chemically generated adduct was compatible with enzyme turnover. The cysteine *S*-thiosulfonate form of TsdA was produced by incubation with tetrathionate, and the excess tetrathionate was removed by desalting. The modified enzyme exhibited no loss of thiosulfate:ferricyanide oxidoreductase activity relative to a control sample that had not been treated with tetrathionate. ESI-MS confirmed that the cysteine *S*-thiosulfonate was stable for the length of time taken to assay the enzyme following desalting. Thus, *S*-thiosulfonation of Cys^123^ is compatible with catalysis as expected if this species is an intermediate in the TsdA reaction. By contrast, *S*-carboxymethylation of Cys^123^ using iodoacetate abolished TsdA activity. This excluded the possibility that enzyme activity is insensitive to the modification state of Cys^123^. Further presumptive evidence that the cysteine *S*-thiosulfonate group is compatible with turnover comes from the observation that the addition of thiosulfate to the *S*-thiosulfonate adduct formed by treatment with equimolar tetrathionate results in heme reduction as judged by the increase in the 553 nm/524 nm peak height ratio and loss of intensity of the band at 637 nm (Fig. 2*A*).

If the *S*-thiosulfonate group is a TsdA reaction intermediate, then it should be removed from the active site cysteine by catalysis (Reaction 6). We tested whether this was the case using iodoacetate to reveal the appearance of the unmodified cysteine residue. We prepared the *S*-thiosulfonate form of TsdA and compared its thiosulfate:ferricyanide oxidoreductase activity in the presence or absence of iodoacetate. If catalysis removes the *S*-thiosulfonate group, then Cys^123^ will be accessible to alkylation for some proportion of the catalytic cycle, and the enzyme will be inhibited by the resulting *S*-carboxymethylation of Cys^123^. A turnover-dependent inhibition of TsdA activity in the presence of iodoacetate is, indeed, observed (Fig. 2*D*). Thus, the *S*-thiosulfonate group is removed from Cys^123^ by catalysis.

Taken together, the experiments described above support the proposal that Cys^123^ *S*-thiosulfonate is an intermediate in the TsdA reaction.

##### Structure of TsdA

**FIGURE 3.**
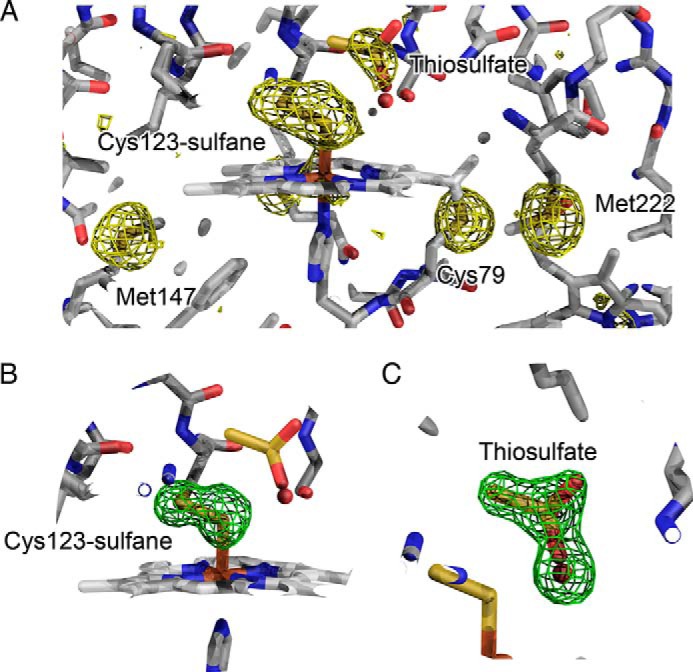
**A sulfane modification on Cys^123^ and a thiosulfate molecule in the active site of TsdA crystallized in the presence of tetrathionate.** *A*, residual log likelihood gradient maps in data collected at λ = 1.4 Å after modeling the contribution of the iron atom. The maps, contoured at 3σ (*gold*), reveal density around the sulfur atoms in the active site. The peak heights correlate well with the thermal mobility and occupancies of the atoms. *B*, simulated annealing omit map, contoured at 3σ (*green*), calculated following exclusion of the two sulfur atoms within the cysteine *S*-sulfane moiety at the active site. *C*, simulated annealing omit map, contoured at 3σ (*green*), calculated following exclusion of both the bound thiosulfate molecule (modeled at 46% occupancy) and the water molecule that occupies the same location when the thiosulfate molecule is not present (54% occupancy).

To gain structural insight into the mechanism of TsdA catalysis, we crystallized the *A. vinosum* enzyme both in the native state and under conditions that were expected to generate the cysteine *S*-thiosulfonate intermediate. Structures were obtained for the enzyme as purified, for the purified cysteine *S*-thiosulfonate adduct, or after co-crystallization with the reductant DTT, with tetrathionate, or with thiosulfate. Although the structures were determined to high resolution (in the range 1.1–2.3 Å) it was difficult in most cases to unambiguously fit the electron density associated with Cys^123^ due to the presence of multiple adducts and/or conformational heterogeneity. However, for TsdA co-crystallized with tetrathionate, the electron density in the active site could be confidently fitted, and this is the TsdA structure presented here. All other structures were identical to this structure outside the active site. The presented structure was determined to 1.3 Å resolution from crystals grown at pH 4.6. In this structure, Cys^123^ is present as a cysteine *S*-sulfane conjugate rather than the cysteine *S*-thiosulfonate adduct expected from reaction with tetrathionate. This interpretation of the active site was confirmed using anomalous scattering in a long wavelength data set (λ = 1.4 Å; Table 1) and simulated annealing omit maps (Fig. 3, *A* and *B*). Experiments in solution show that the thiosulfonated form of TsdA is unstable and breaks down over time to form cysteine *S*-sulfane, which we could trap with iodoacetate as a cysteine *S*-sulfane-carboxymethyl adduct (Fig. 2*E*). This observation provides a possible explanation for the presence of cysteine *S*-sulfane in the crystallized enzyme. In an attempt to overcome the instability of the cysteine *S*-thiosulfonate intermediate, we tried to generate the intermediate directly in native TsdA crystals by soaking with either tetrathionate or thiosulfate before cryo-cooling. However, this approach was unsuccessful, with little Cys^123^ derivatization observed.

**TABLE 1:** **X-ray data collection, phasing, and refinement statistics**

**Data collection**		
X-ray source (λ)	Diamond, i03 (0.920 Å)	Diamond, i03 (1.40 Å)
Space group	C2	C2
Cell dimensions	*a* = 79.28 Å, *b* = 70.42 Å, *c* = 57.91 Å	*a* = 79.37 Å, *b* = 70.37 Å, *c* = 57.95 Å
	α = 90.00°, β = 129.34°, γ = 90.00°	α = 90.00°, β = 129.39°, γ = 90.00°
Resolution (Å)	44.52-1.29 (1.32-1.29)*^a^*	21.72-1.66
Total reflections	170,161	618,046
No. of unique reflections	60,432	28,874
Completeness (%)	97.8 (98.0)	97.2 (87.5)
Multiplicity	2.8	21.4
*R*_merge_	0.034 (0.47)	0.068 (0.70)
*I*/δ(*I*)	15.5 (2.3)	32.4 (4.1)

**Refinement statistics**		
*R* (%)	14.83 (24.70)	
*R*_free_ (%)	16.40 (26.40)	

**Root mean square deviation from idealized covalent geometry**		
Bond length (Å)	0.03	
Bond angles (degrees)	2.42	
Average *B* value (Å^2^)	22.52	
Ramachandran outliers (%)	0.42	
Residues modeled	234	
Non-protein molecules	202 waters, 2 hemes, 1 thiosulfate	
Molprobity score	1.22

*^a^* Information in parenthesis refers to the last resolution shell.

**FIGURE 4.**
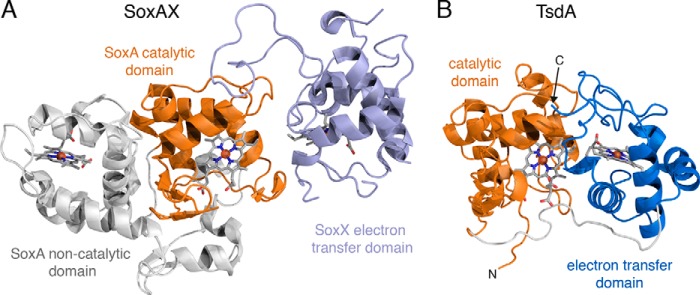
**Structural comparison between *R. sulfidophilum* SoxAX (*A*) and *A. vinosum* TsdA (*B*).** The proteins are displayed with their catalytic domains (*orange*) in the same orientation as determined by an alignment of their C_α_ atoms. The hemes are shown in *stick representation* with carbon *gray*, nitrogen *blue*, oxygen *red*, sulfur *yellow*, and iron *orange*.

**FIGURE 5.**
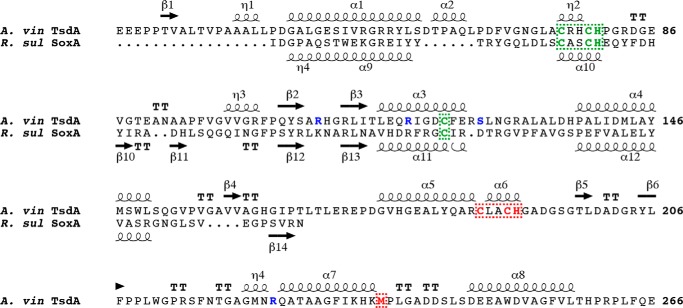
**The sequence and secondary structure of *A. vinosum* TsdA.** For comparison, the catalytic domain of *R. sulfidophilum* SoxA is also shown. Secondary structures are depicted using *coils* for α- and η-helices, *arrows* for β-strands, and *T* for β-turns. Residues involved in binding either the heme irons or heme vinyl groups are indicated in *green* for the catalytic domain heme and in *red* for the electron transfer domain heme. The TsdA active site residues substituted in this work are *colored blue*. The TsdA signal peptide sequence is not included in the figure.

TsdA comprises two cytochrome *c*-like domains connected by a long unstructured linker (Figs. 4*B* and 5). As predicted (7), the N-terminal domain is structurally homologous to SoxA family cytochromes (Fig. 4*A*) and can be aligned with the catalytic domain of the *R. sulfidophlium* SoxA protein (residues 177–282) with a root mean square deviation of 1.15 Å over 45 equivalent Cα atoms. The C-terminal domain of TsdA has a standard mitochondrial cytochrome *c* family fold and exhibits the highest structural similarity to the C-terminal domain of nitrite reductase from *P. haloplanktis* (alignment of root mean square deviation of 2.47 Å over 55 equivalent Cα atoms with PDB entry 2ZOO).

**FIGURE 6.**
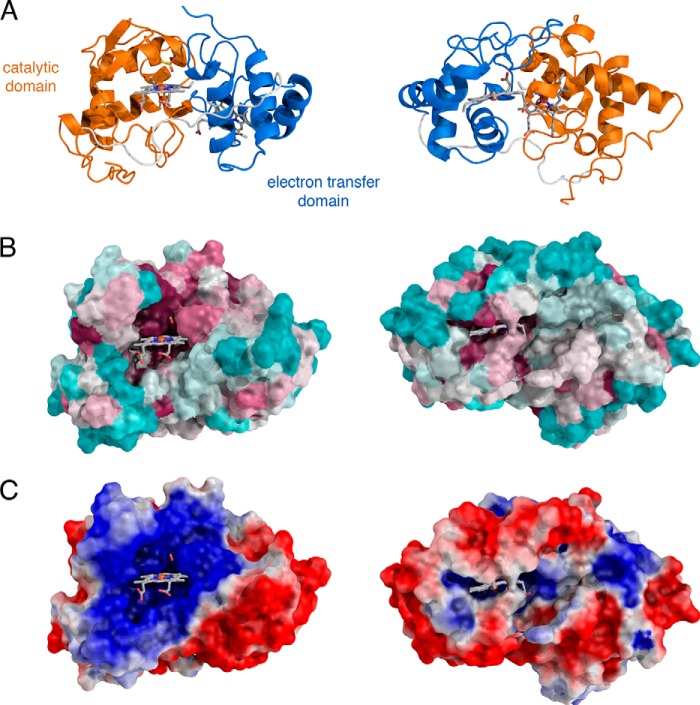
**Accessibility and environment of the TsdA hemes.** The *views* in each *column* are in the same orientation. The *left-hand panels* show the entrance to the catalytic site, and the *right-hand panels* show the environment around the electron acceptor site. The hemes and bound thiosulfate are shown in *stick representation* using the same *color scheme* as Fig. 4. *A*, schematic representation of the protein backbone. *B*, surface conservation calculated using Consurf (40) from 150 sequences verified as TsdA proteins by homology across the full polypeptide length, including the conservation of the two type *c* heme motifs, the heme axial ligand residues, and Arg^119^. *Cyan*, regions of lowest conservation; *magenta*, regions of highest conservation. *C*, the surface potential of TsdA calculated using APBS (41) *colored* from positive (*blue*) to negative (*red*). Note that the calculations do not include the heme molecules.

Each cytochrome domain contains one covalently bound heme molecule (Figs. 4*B* and 5). The heme iron of the N-terminal domain heme is coordinated by the sulfane atom of the Cys^123^-*S*-sulfane conjugate and by His^80^. The ligands to the heme in the C-terminal domain are Met^236^ and His^191^. The C-terminal domain heme exhibits significant distortion from planarity, whereas the N-terminal domain heme shows far less distortion. Such heme “ruffling” is observed for all *c*-type cytochromes, but the functional significance is not well understood (33). The packing of the two domains positions the two hemes close together, resulting in a nearest heme macrocycle edge-to-edge distance of 8.1 Å. This separation would allow very rapid interheme electron transfer (34). Fast electron movement between the hemes may be important for TsdA catalysis because the oxidation of thiosulfate releases two electrons, but each heme can accept only one electron. Both hemes are partially accessible to the solvent surrounding the enzyme. In the N-terminal domain, a channel provides substrate access to the Cys^123^ side of the heme, where catalysis is assumed to occur (Fig. 6, *B* and *C*). The environment both within the channel and on the protein surface surrounding the channel is highly basic (Fig. 6*C*), suggesting that electrostatic funneling is used to attract the anionic substrate molecules into the active site. In the C-terminal domain, the heme iron is buried, but one edge of the heme macrocycle lies close to the molecular surface and is exposed to solvent through a cleft (Fig. 6, *B* and *C*). The structural environment of the two hemes suggests a model for electron transfer in TsdA in which the electrons derived from thiosulfate oxidation are initially accepted by the heme in the N-terminal “catalytic” domain before being passed to the heme in the C-terminal “electron transfer” domain and then to an external electron acceptor protein positioned on the surface of TsdA near the exposed heme edge. The TsdA surface around the exposed edge of the electron transfer heme shows higher sequence conservation than the bulk of the protein surface, suggesting that TsdA provides a specific binding site for its redox partner (Fig. 6*B*).

A comparison of the structures of TsdA and SoxAX shows that each packs the same face of the catalytic domain against their respective electron transfer domains (Fig. 4). The relative orientation of the two domains is different in the two proteins, resulting in a longer heme macrocycle edge-to-edge distance in *R. sulfidophilum* SoxAX (10.7 Å) than in TsdA (8.1 Å). However, this difference is unlikely to be mechanistically important because in both enzymes electron transfer between the hemes will be rapid relative to catalysis.

##### Active Site Structure

**FIGURE 7.**
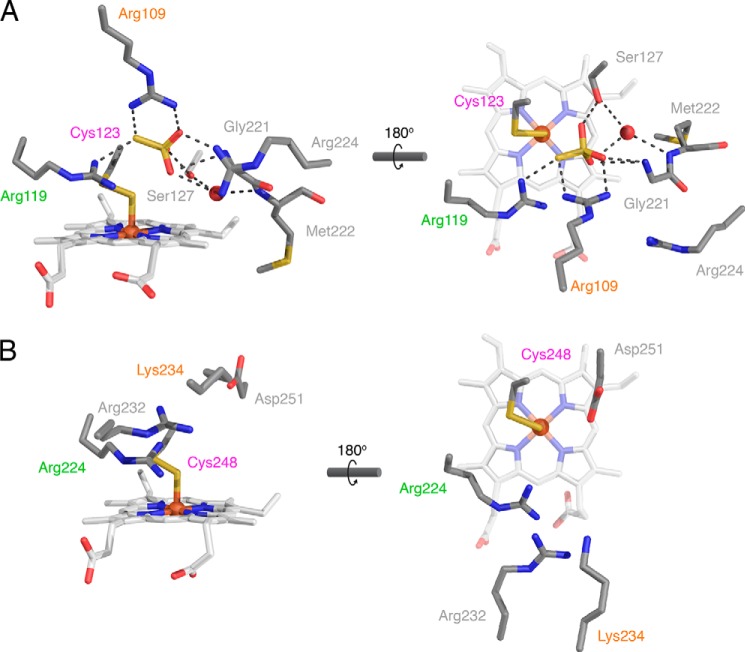
**Comparison of the active sites of TsdA and SoxAX.** Active sites are viewed from approximately the same orientations as in Fig. 6 (*left-hand panels*) and Fig. 4 (*right-hand panels*). Amino acid side chains, hemes, and thiosulfate are shown in *stick representations* and *colored* as in Fig. 4. Ordered water molecules are shown as *red spheres*. Non-covalent bonding interactions are indicated with *black dotted lines*. Structurally homologous amino acids are *labeled* in the same *color. A*, active site of *A. vinosum* TsdA. Crystals were grown in the presence of tetrathionate, and a thiosulfate molecule is present in the active site. Note that the *right-hand* heme propionate exhibits an additional minor conformer (not shown) in which the propionate points *upward* into the active site. *B*, active site of *R. sulfidophilum* SoxAX (PDB entry 1H32). Note that for consistency with TsdA and the current PDB numbering convention, the SoxA amino acids are *numbered* from the start of the precursor protein and thus differ from the numbering scheme used previously (9).

The active site of TsdA lies on the Cys^123^ side of the catalytic heme. The active site cavity is considerably smaller than the one found in SoxAX, reflecting the need for the SoxAX active site to accommodate a large peptide co-substrate. In SoxAX, the active site cavity is formed exclusively from residues in the catalytic domain. However, in TsdA, residues from the electron transfer domain intrude into one side of the active site (Fig. 7*A*), and it is this insertion that is primarily responsible for the reduced active site volume in TsdA relative to SoxAX.

The active site of TsdA co-crystallized with tetrathionate contains density that is well interpreted as a molecule of thiosulfate at 46% occupancy (Figs. 3, *A* and *C*, and 7*A*). The presence of thiosulfate in the crystallization environment is expected because it is formed as a product of the initial thiosulfonation of Cys^123^ by tetrathionate (Reaction 6). Thiosulfate is both the substrate of TsdA and an analogue of the Cys^123^ *S*-thiosulfonate intermediate. Thus, the thiosulfate molecule seen in the crystal structure provides insight into the way TsdA binds substrates and intermediates. The thiosulfate molecule has bonding interactions with the amino acid side chains of Arg^109^, Arg^119^, and Ser^127^ as well as with the main chain amide group of Gly^221^ from the electron transfer domain. Arg^119^ is invariant in TsdA and SoxA proteins (7). Arg^109^ is less well conserved, being replaced by a lysine in some TsdA molecules and by either lysine or serine in SoxA proteins. Ser^127^ is invariant in TsdA proteins but is not present in SoxA. Examination of the available SoxAX structures shows that an additional arginine side chain (Arg^232^ in *R. sulfidophilum* SoxA) is present between the Arg^109^ and Arg^119^ equivalents in SoxA (Fig. 7*B*). This arginine residue is invariant in SoxA proteins and would be well placed to coordinate a thiosulfate molecule at a position comparable with that seen in the TsdA structure (Fig. 7*A*).

A final residue of potential catalytic interest in the TsdA active site is Arg^224^, which is located on the electron transfer domain (Fig. 7*A*). Although Arg^224^ is not involved in coordinating the thiosulfate molecule found in the crystal structure, it still has the potential to form bonding interactions with the negatively charged substrates and intermediate. Arg^224^ is partially conserved in TsdA proteins, but no equivalent is present in the SoxA active site.

##### Functional Importance of Active Site Residues

The crystal structure of TsdA identifies active site residues Arg^109^, Arg^119^, Ser^127^, and Arg^224^ as potentially involved in substrate binding and catalysis. We investigated the importance of these residues in the TsdA mechanism by characterizing corresponding alanine-substituted variants. Because the kinetics of the TsdA reaction with ferricyanide as the oxidant are complex, with the apparent *K_m_* for thiosulfate decreasing as the electron acceptor concentration decreases (6), we chose to assess the steady state kinetics of the TsdA variants at both a higher (1 mm) and lower (0.2 mm) ferricyanide concentration (Table 2).

**TABLE 2:** **Kinetic analysis of TsdA variants** Activity measurements were carried out at pH 4.25 with either 0.2 mm or 1 mm potassium ferricyanide as electron acceptor. *n* is the apparent Hill coefficient. Error values show the S.E. for fits of curves with duplicate points.

Variant	0.2 mm K_3_Fe(CN)_6_			1 mm K_3_Fe(CN)_6_		
	*K_m_*	*k*_cat_	*n*	*K_m_*	*k*_cat_	*n*
	*mm S_2_O*_*3*_^*2*−^	*s*^−*1*^		*mm S_2_O*_*3*_^*2*−^	*s*^−*1*^	
Wild type	0.22 ± 0.03	920 ± 50	1.4 ± 0.25	2.2 ± 0.3	4280 ± 150	1.52 ± 0.25
S127A	4.4 ± 0.5	1530 ± 150	2.4 ± 0.7	10.7 ± 6.2	3060 ± 920	1.1 ± 0.4
R119A	17 ± 2	378 ± 20	1.2 ± 0.1	47 ± 7	640 ± 40	1.0 ± 0.05
R109A	1.11 ± 0.25	780 ± 95	1.6 ± 0.5	5.8 ± 0.8	3210 ± 310	1.7 ± 0.3
R224A	0.44 ± 0.05	870 ± 50	1.3 ± 0.2	2.5 ± 0.8	3670 ± 460	1.14 ± 0.26

**FIGURE 8.**
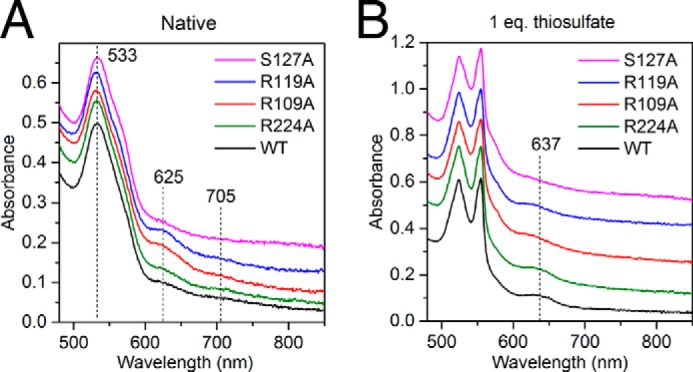
**Visible spectral characteristics of TsdA active site variants.** WT or the indicated single amino acid variants of TsdA were analyzed at a concentration of 25 μm in 100 mm ammonium acetate, pH 4.25. Variant spectra are offset for clarity. Samples were either left untreated (*A*) or incubated for 3 min (*B*) with equimolar thiosulfate.

**FIGURE 9.**
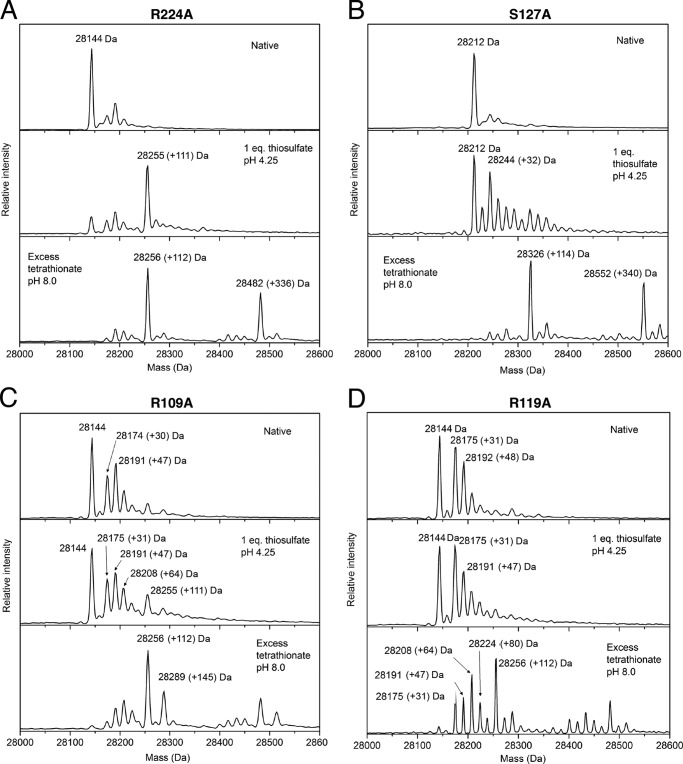
**Characterization of the active site cysteine adducts formed by TsdA variants.** The indicated TsdA variants (*A–D*) were either left untreated (*Native*), incubated with equimolar thiosulfate for 3 min in 100 mm ammonium acetate, pH 4.25, or incubated with 50 mm tetrathionate for 1 h in 30 mm Tris-HCl, pH 8.0, 160 mm NaCl. Samples were quenched with 0.1% TFA and analyzed by ESI-MS. Note that the tetrathionate-treated samples contain a duplicated series of peaks at masses 340 Da greater than those of the primary set of peaks arising from proteins in complex with a tetrathionate molecule.

The R224A variant exhibited thiosulfate oxidation kinetics that were not significantly different from those of the parental enzyme except for an increase in *K_m_*(thiosulfate) under the lower ferricyanide assay condition (Table 2). The variant also resembled the parental TsdA protein in its ability to form the *S*-thiosulfonate derivative with either thiosulfate or tetrathionate (Figs. 8*B* and 9*A*). These data suggest that Arg^224^ does not play a substantive role in the TsdA mechanism. The other three variants all showed perturbed thiosulfate oxidation kinetics relative to the parental enzyme confirming their involvement in TsdA catalysis (Table 2). The R109A variant shows small decreases in *k*_cat_ for thiosulfate oxidation relative to the parental enzyme and a moderate increase in *K_m_*(thiosulfate), whereas the S107A variant exhibits little change in *k*_cat_ but a more significant increase in *K_m_*(thiosulfate). The R119A variant shows larger changes in both kinetic parameters than either of the other variants. Thus, substitution of the most conserved residue has the largest effect on reaction kinetics. The TsdA reaction involves two thiosulfate molecules, so the substrate concentration *versus* rate curve for TsdA has been interpreted as showing positive cooperativity using the Hill equation (6). Some of the TsdA active site substitutions characterized here alter the apparent Hill coefficient for the thiosulfate oxidation reaction (Table 2), but these changes are at the margins of significance, so we do not offer mechanistic interpretations.

The three activity-perturbed TsdA variants differ in the amount of high spin heme they contain, as judged by the size of the 625 nm peak in the visible spectrum (Fig. 8*A*). The R109A and R119A variants have substantially more of this species than the wild type enzyme, and the S127A variant has considerably less. ESI-MS of these variants shows that a proportion of the R109A and R119A molecules are present in modified forms with mass peaks at +32, +47, and +64 Da (Fig. 9, *C* and *D*). Because these modifications are likely to be located on the Cys^123^ active site heme ligand, they may explain the higher high spin heme content of these variants. Identification of the modifications seen in these variants is hampered by the mass equivalence between a sulfur atom (32 Da) and two oxygen atoms (2 × 16 Da). Nevertheless, the presence of modified forms of TsdA suggests that the two variants are more prone than the wild type enzyme either to oxidation of Cys^123^ or to adventitious reactions with sulfur compounds present during cell growth.

All three variants were reduced by equimolar thiosulfate at pH 4.25 (Fig. 8*B*), consistent with their still appreciable thiosulfate dehydrogenase activity (Table 2). The variants do not, however, develop the prominent 637 nm band seen for the wild type enzyme, which is assigned to S-thiosulfonation of Cys^123^ (Figs. 2*B* and 8*B*). ESI-MS analysis was used to probe the species formed when the variants react with thiosulfate. In all three cases, a significant proportion of the variant remained unmodified (Fig. 9, *B–D*), in contrast to the behavior of the wild type enzyme (Fig. 2*C*), and only a small peak is present at the mass corresponding to cysteine *S*-thiosulfonate (+111 Da). The R109A and R119A variants still exhibit a series of prominent peaks with masses between those of the thiosulfonated and underivatized forms of the enzyme. Similar mass peaks are now also seen for the S127A variant. Because each variant was observed to be reduced by thiosulfate (Fig. 8*B*), we infer that the Cys^123^ *S*-thiosulfonate adduct is still formed but is chemically unstable at pH 4.25 in the absence of key interactions provided by the substituted amino acids.

As an alternative way of investigating the stability of the cysteine *S*-thiosulfonate adduct in the TsdA variants, we generated the adduct chemically through exposure to a high concentration of tetrathionate over an extended period at pH 8, as described earlier (Fig. 1). Under these conditions, the three activity-affected TsdA variants all formed the *S*-thiosulfonated species, although the R119A variants showed additional lower mass peaks (+64 and +80 Da), and the R109A variant showed a higher mass peak (+145 Da, perhaps corresponding to Cys-S-S-SO_3_^−^) (Fig. 9, *B–D*). Taken together, these data suggest that the active site residues Arg^109^, Arg^119^, and Ser^127^ influence the reactivity of Cys^123^ and the stability of its modifications.

## DISCUSSION

The SoxA family of cysteine-ligated cytochromes catalyze the oxidative conjugation of a thiosulfate molecule to another thiolate or, in some cases, the reverse of this reaction. It has been suggested that the heme cysteine ligand participates in the catalytic chemistry of these enzymes by forming a covalent linkage to the thiosulfate molecule, which is then resolved by disulfide exchange with an incoming thiolate (7, 9). However, experimental evidence in support of this model has been difficult to obtain.

Using the SoxA family enzyme TsdA thiosulfate dehydrogenase as our model system, we have now made a series of observations that support the proposal that a thiosulfonate adduct of the cysteine heme ligand is a catalytic intermediate in this enzyme family. First, the cysteine is chemically reactive and is accessible to modification by electrophiles, as required when the enzyme operates in the reverse direction. Second, the cysteine is modified to an *S*-thiosulfonate adduct by exposure to one equivalent of either thiosulfate or tetrathionate, as predicted for the forward and reverse half-reactions, respectively. Third, the activity of TsdA is unimpeded by *S*-thiosulfonation of the cysteine, as expected if this species is a catalytic intermediate. Fourth, a thiosulfonate group placed on the cysteine is removed during enzyme turnover, as predicted if this adduct is an intermediate in the reaction.

Kilmartin *et al.* (11) have reported that the SoxAX enzyme from *Starkeya novella* still has catalytic activity after the active site cysteine residue has been removed. This observation is difficult to understand in the light of the experiments presented here as well as earlier data showing an essential functional role for the equivalent cysteine in TsdA (7). Kilmartin *et al.* (11) have proposed an alternative SoxA mechanistic model that does not rely on the active site heme cysteine ligand but is instead based on catalysis by a copper ion detected in *S. novella* SoxAX preparations by EPR spectroscopy (8, 11, 35). However, structural biology provides no support for this model because neither copper nor a plausible copper binding site is present in any of the structures of SoxA family enzymes now available, including that of *S. novella* (11).

We obtained the crystal structure of TsdA with a thiosulfate molecule bound in the active site and this has provided additional insight into SoxA family catalysis. The thiosulfate molecule is coordinated by three active site residues that are conserved to a greater or lesser extent in TsdA and SoxA proteins. Amino acid substitutions confirmed the importance of these residues in TsdA catalysis. Alanine-substituted variants at two of these positions exhibited partial modification of the cysteine heme ligand, suggesting that these residues not only assist productive catalysis but also function to suppress inappropriate reactivity at the active site cysteine.

In the crystal structure, the active site cysteine residue has been modified to an *S*-sulfane derivative that coordinates the heme iron through the sulfane atom (Fig. 7*A*). An identical modification of the active site cysteine has been observed in multiple SoxAX structures, where it has been speculated to be either an off-pathway breakdown product of the cysteine *S*-thiosulfonate intermediate or a purification artifact (9–11). Our MS data strongly support the proposal that the sulfane derives from decomposition of the thiosulfonated intermediate. The active site cysteine residue is unmodified in purified TsdA (Fig. 1*A*), but once the enzyme has been thiosulfonated *in vitro*, the sulfane derivative appears over time (Fig. 2*E*). The same process probably occurred in the TsdA crystals because these were grown in the presence of tetrathionate and so would initially contain thiosulfonated protein. Earlier EPR data suggest that the active site cysteine thiolate directly ligates the active site heme iron (7), and it would certainly be structurally plausible for the cysteine sulfur atom to be located at the heme axial position occupied by the sulfane atom in the crystal structure.

**FIGURE 10.**
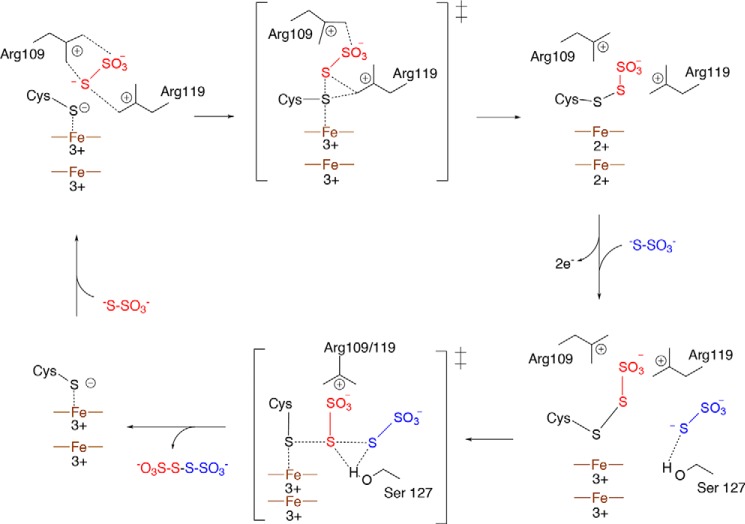
**A possible reaction mechanism for TsdA.** The first thiosulfate molecule (*red*) is positioned by Arg^109^ and Arg^119^, and the same residues stabilize the cysteine *S*-thiosulfonate group once this has formed. Following heme reoxidation by an external electron acceptor, the sulfane atom of the second thiosulfate molecule (*blue*) attacks the thiosulfonate group in a thiol-disulfide exchange reaction. In the transition state, the leaving group (Cys^123^) is stabilized by the catalytic heme iron(III), and the transition state is stabilized by Ser^127^. The *top* heme is the catalytic heme, and the *bottom* heme is the electron transfer heme.

Combining the biochemical evidence for a cysteine *S*-thiosulfonate intermediate with the active site substitution analysis, we propose the mechanistic model for TsdA shown in Fig. 10. The first half-reaction in the proposed TsdA mechanism (Reaction 5) has considerable analogy to the mechanism of *de novo* disulfide bond formation catalyzed by enzymes with a FAD or quinone cofactor, such as dihydrolipoamide dehydrogenase or the DsbB protein of the bacterial protein disulfide bond-forming machinery (36, 37). In these enzymes, the electrophilic thiol group is activated through formation of a charge transfer complex with the organic cofactor, and the cofactor receives the two electrons released in the disulfide bond-forming reaction (37, 38). In a similar way, the active site heme in TsdA draws electrons away from the catalytic cysteine, whereas the heme pair functions as the two-electron acceptor for disulfide bond synthesis. The close separation of the two TsdA hemes would allow rapid removal of both electrons from the reactive sulfur atom and thus limit the lifetime of any one electron-oxidized radical state.

Both half-reactions in the TsdA reaction involve disulfide bond formation with an incoming thiosulfate nucleophile. The thiosulfate molecule in the crystal structure plausibly occupies the position of the thiosulfate molecule in the first half-reaction because only a small movement of the thiosulfate would be needed to achieve the geometry expected in the disulfide bond-forming transition state: the attacking sulfur atom (thiosulfate sulfane atom) in line with the bond between the target sulfur atom (heme-ligating sulfur atom) and the leaving group (the heme iron) (39) (Fig. 7*A*). It is likely that, once formed, the cysteine *S*-thiosulfonate intermediate remains coordinated to the conserved basic residues that bind the thiosulfate molecule. Based on accessibility and space considerations, the thiosulfate molecule required in the second half-reaction would probably be located roughly where the Ser^127^-coordinated water molecule is found in the crystal structure (Fig. 7*A*). Such a location also makes sense from the point of view of the differing substrates that TsdA and SoxA use in the second half-reaction (Reactions 3 and 6). This is because Ser^127^ is conserved only in TsdA enzymes and might therefore be expected to interact with the TsdA-specific substrate involved in the second half-reaction and because this side of the active site cavity is enlarged in SoxA enzymes, as would be necessary to accommodate the SoxY carrier arm that acts as substrate in the second SoxA half-reaction.

Completion of the catalytic cycle requires reoxidation of the TsdA hemes. Does this process affect the chemistry occurring at the active site? We found that the addition of excess thiosulfate to TsdA in the absence of an external electron acceptor resulted in enzyme that was both reduced and *S*-thiosulfonated (Fig. 2, *B* and *C*). This suggests that catalysis under these conditions is paused after the first half-reaction (Reaction 5) and, consequently, implies that the second half-reaction is gated by heme reoxidation. A possible mechanistic explanation for this phenomenon is that the ferric state of the catalytic heme would help stabilize the thiolate form of Cys^123^ formed by release of tetrathionate in the second half-reaction (Fig. 10).

## Footnotes

6 The abbreviations used are:

TsdA: thiosulfate dehydrogenase
ESI-MS: electrospray ionization mass spectrometry.
